# Differentiation of different stages of brain tumor infiltration using optical coherence tomography: Comparison of two systems and histology

**DOI:** 10.3389/fonc.2022.896060

**Published:** 2022-08-30

**Authors:** Paul Strenge, Birgit Lange, Wolfgang Draxinger, Christin Grill, Veit Danicke, Dirk Theisen-Kunde, Christian Hagel, Sonja Spahr-Hess, Matteo M. Bonsanto, Heinz Handels, Robert Huber, Ralf Brinkmann

**Affiliations:** ^1^ Medical Laser Center Luebeck, Luebeck, Germany; ^2^ Institute of Biomedical Optics, University of Luebeck, Luebeck, Germany; ^3^ Institute for Neuropathology, University Medical Center Hamburg-Eppendorf, Hamburg, Germany; ^4^ Department of Neurosurgery, University Medical Center Schleswig-Holstein, Luebeck, Germany; ^5^ Institute of Medical Informatics, University of Luebeck, Luebeck, Germany; ^6^ German Research Center for Artificial Intelligence, Luebeck, Germany

**Keywords:** brain, tumor, glioblastoma multiforme, OCT, neural network, attenuation (absorption) coefficient, optical coherence tomography

## Abstract

The discrimination of tumor-infiltrated tissue from non-tumorous brain tissue during neurosurgical tumor excision is a major challenge in neurosurgery. It is critical to achieve full tumor removal since it directly correlates with the survival rate of the patient. Optical coherence tomography (OCT) might be an additional imaging method in the field of neurosurgery that enables the classification of different levels of tumor infiltration and non-tumorous tissue. This work investigated two OCT systems with different imaging wavelengths (930 nm/1310 nm) and different resolutions (axial (air): 4.9 μm/16 μm, lateral: 5.2 μm/22 μm) in their ability to identify different levels of tumor infiltration based on freshly excised *ex vivo* brain samples. A convolutional neural network was used for the classification. For both systems, the neural network could achieve classification accuracies above 91% for discriminating between healthy white matter and highly tumor infiltrated white matter (tumor infiltration >60*%*) .This work shows that both OCT systems with different optical properties achieve similar results regarding the identification of different stages of brain tumor infiltration.

## 1 Introduction

Each year, more than 700,000 patients are diagnosed with different tumor types of the central nervous system worldwide, which need to be surgically removed (1). Particularly in glioma and metastasis surgery, the neurosurgeon faces the challenge of removing as many tumors as possible because the survival of a patient directly correlates with the extent of tumor resection (2). To achieve this and spare healthy brain tissue, brain tissue must be identified, which is still a major challenge for the surgeon. This challenge is aggravated by the infiltrative growth of glioblastoma multiforme, which results in ill-defined tumor borders. Glioblastoma multiforme makes up 15 to 20% of all primary brain tumors and is classified by the World Health Organization as a grade IV tumor (3–5). Glioblastoma multiforme is characterized by aggressive and invasive growth, which leads to a median survival time of three months if no treatment is performed (6). At present, the conventional surgery set-up, with intraoperative surgical navigation, including surgical microscopes and intraoperative magnetic resonance imaging (MRI), cannot properly visualize the gradual decrease in the tumor cell density at the infiltration zone to the surgeon. For example, Kut et al. showed that a surgeon with the described surgical set-up reached 100% sensitivity and specificity of between 40 and 50% for the identification of brain tumors (7).

These reasons motivated research on intraoperative optical coherence tomography (OCT) as an additional imaging modality as early as 2006 (8). OCT is an imaging method that provides tomographic images by the interference of low-coherent light (9, 10). OCT is well established in ophthalmology [e.g., retinal imaging (11)], because data are acquired fast, contactless and with a resolution of a few micrometers. The characteristics of OCT images are similar to ultrasound images and are difficult to interpret by the neurosurgeon during an intraoperative setting. Therefore, multiple research groups have successfully demonstrated different ways to process the data for the surgeons to identify various brain tissue types.

One approach exploits optical tissue parameters. Schmitt et al. and Faber et al. introduced methods to extract the attenuation coefficient, which describes the exponential decay of the OCT signal along the depth axis (12–14). The method was further developed by Vermeer et al., Yuan et al., and Turani et al. (15–17). The tumor classification based on the optical parameters assumes that the tumor changes the optical properties of the tissue, which has already been described by Böhringer et al. in 2009 (18). In their study, *ex vivo* and *in vivo* OCT data were acquired from 15 selected brain regions of nine patients, which contained healthy brain tissue and glioblastoma multiforme, among others. It was found that the attenuation profile of the OCT signal varies depending on whether the tissue is healthy or tumorous. These first findings were further elaborated by Kut et al. and Yashin et al., who showed that the attenuation coefficient for white matter decreases with increasing tumor infiltration (7, 19). Yashin et al. hypothesized a decrease with the degradation of the myelin fibers, which are present in healthy white matter but break down with increasing tumor invasion. Both groups could successfully classify healthy white matter tissue and tumor tissue.

Another approach considers the usage of structural information. Böhringer et al. stated that structural heterogeneities occur in tumorous tissue, while healthy brain tissue has structural homogeneity (18). Various groups have applied different methods to extract the structural information for the classification of brain tumor tissue. For example, Lenz et al. used a combination of texture features like Haralick’s texture features combined with a principal component analysis (PCA) and a support vector machine approach (SVM) to differentiate *ex vivo* OCT meningioma samples from healthy brain tissue (20). Möller et al. used the same approach successfully for the classification of brain metastases (21). Moisseev et al. used patches of the OCT signal, acquired from mouse *ex vivo* OCT B-scans, which were subsequently decomposed *via* PCA for creating feature vectors (22). In their study, Gesperger and Lichtenegger et al. used OCT images from *ex vivo* human brain samples, which in contrast to the other publications, were acquired by a high resolution optical coherence microscope, to train a neural network (23). The high resolution of the OCT system (lateral resolution of 1.8 μm, axial resolution of 0.88 μm) enhances the structural differences between the different tissue types. All these approaches achieved a classification accuracy of over 90%. However, different OCT parameters (wavelength, spatial resolution) were used, and some studies were limited to the differentiation between healthy and tumorous white matter. The differentiation of all tissue types (white and gray matter—healthy and tumorous) was investigated only in a few studies (23), (24). In these publications, detailed information on the distribution of white and gray matter in the sample set is missing. It was also shown that the accuracy drops significantly when samples with low tumor infiltration are classified (23).

This work tried to account for these problems by applying a very detailed label to the histological data of brain tissue samples that were imaged immediately after excision. Heterogeneous tissue combinations and different grades of tumor infiltration were considered. The labels were transferred to corresponding OCT B-scans. Each sample was imaged using two OCT systems: one of the OCT systems had an acquisition rate of up to 1.6 MHz, which makes the system suitable for real-time *in vivo* imaging if it is mounted to the surgical microscope, for example (25). This work was used to prove that such a system can classify tumor-infiltrated brain tissue from healthy brain tissue. Additionally, a second OCT system was used during the study as a control system. The two systems differ in their acquisition wavelength and lateral and axial resolution. The use of two OCT systems allowed us to get more insight into which OCT properties guarantee a good tissue classification. It was investigated if, due to different optical properties of the tissue at different wavelengths and structural information, the two OCT systems differ in their ability to identify tumor regions. In the end, neural networks were used to classify healthy brain tissue from tumor-infiltrated tissue based on the *ex vivo* dataset for each OCT system. The neural networks used extracted regions of the B-scans or optical properties like the attenuation coefficient, which were determined from the extracted regions, as input.

## 2 Materials and methods

### 2.1 OCT systems

The data acquisition was performed with two OCT systems: a spectral domain (SD) OCT system (Callisto, Thorlabs Inc.) and a swept source OCT system (Optores GmbH, Germany). The core of the latter system consists of a Fourier domain mode locked (FDML) laser with a central wavelength of 1,310 nm and a spectral bandwidth of 110 nm (26). The FDML technology allowed the acquisition of OCT A-scans at a rate of 1.6 (27). An objective lens with a focal length of 54 and a numerical aperture of 0.021 (LSM04, Thorlabs Inc.) was used during data acquisition. The lateral and axial resolution of the OCT systems were determined through point spread function (PSF) measurements, using the full width at half maximum (FWHM) of nano particles dispersed in a non-scattering medium (OCT Resolution Validation Phantom, National Physical Laboratory) (28). The results gave a lateral resolution of around 22 μm and an axial resolution of 16 μm in air. The scan field of the system was set to 6 mm × 6 mm. The SD-OCT had a central wavelength of 930 nm and a spectral bandwidth of 127 nm. The system was equipped with an objective lens with a focal length of 36 mm and a numerical aperture of 0.051 (LSM03-BB, Thorlabs, Inc.). The lateral and axial PSF measurements showed a lateral and axial resolution of 5.2 μm and 4.9 μm in air. The field of view was 2 mm × 5.2 mm. The system was additionally equipped with a spectator camera, which allowed the acquisition of color images in a range of 12.8 mm × 9.6 mm. Both OCT systems were mounted on a movable rack system in order to be used in a separate room adjacent to the operation theater. The close distance of the OCT system to the tumor extraction assured that the sample was imaged within 15 min after excision and that possible *ex vivo* tissue changes were kept to a minimum (29).

### 2.2 Data acquisition

**Figure 1:**
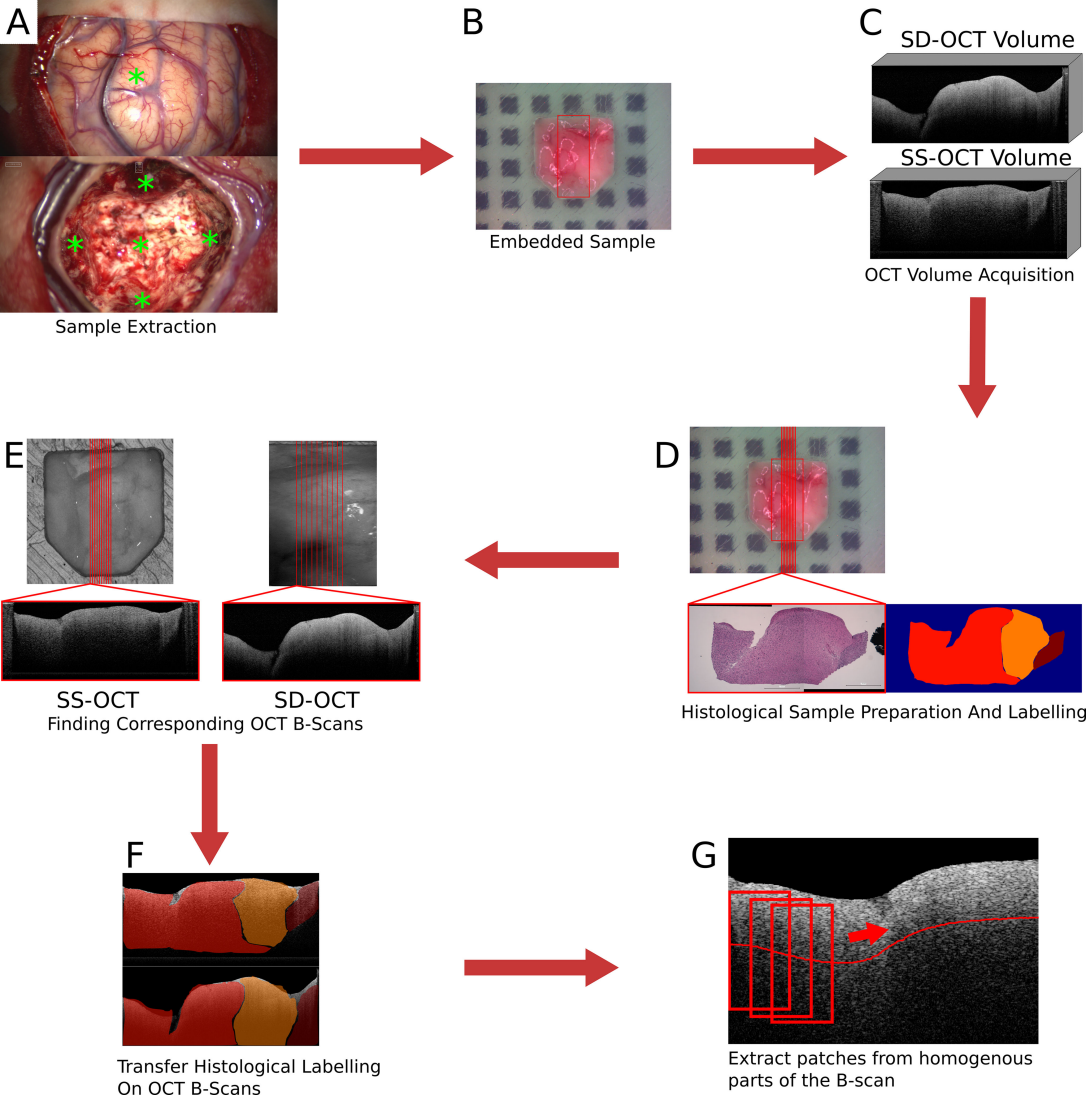
Images of the brain surface and the resection cavity **(A)**. * marks the locations where samples were extracted. Embedded sample in agarose-filled tissue cassette **(B)**. In **(B)**, the red square marks the field of view of the SD-OCT system. Examples of acquired OCT volumes by the two OCT systems **(C)**. An example of the H&E stained histological cut and the label set by the neuropathologist **(D)**. In **(D)**, the location of the histological section was marked by the red line. Extracting the corresponding OCT B-scans from the OCT volumes based on the position of the transformed cutting line **(E)**. An example of the transferred label on the corresponding OCT B-scans **(F)**. Extraction of patches from homogeneous parts of the B-scan **(G)**.

Over the course of the clinical study (Study No.: 18-204, ethics committee University of Luebeck) 21 patients contributed samples. For an overall 73 samples of 15 patients, complete datasets were acquired with both OCT systems (see **Table 1**). Seven of these patients were diagnosed with glioblastoma multiforme (WHO IV), four were classified as WHO III and WHO II, and the remaining four as metastasis (5). Samples were excised from the untouched brain surface, from tissue above the main tumor mass, the main tumor mass, and from the border of the resection cavity after the resection was finished by the surgeon (**Figure 1A**). Samples were not taken if the location was too close to a functional area. After the excision, each sample was embedded in an agarose-filled tissue cassette (**Figure 1B**) (30). The tissue cassette had four imprints of different sizes (3x3x1, 4x4x2, 5x5x3 and 6x6x3 mm^3^). The sample was placed into one of these imprints, depending on the sample size and which shape it matched the best. Over the course of the data acquisition, the soft brain sample will take over the predefined shape of the imprint. The shape of the sample functions as *a priori* information for the transformation of the histological information and as a measure to control the cutting process. Each OCT system acquired one OCT volume of the sample within a 15-minute time frame after the sample extraction (**Figure 1C**). Afterwards, the sample was fixed with a 4.5% formalin solution for at least 24 h before being processed further by the neuropathology. Fixation with formalin also fixed the shape of the sample. Ten hematoxylin and eosin (H&E) stained histological sections were cut equidistantly (100 μm) from each sample. The position and orientation of the sections were defined prior to the cutting by cutting lines on the top view image of the sample (**Figure 1D**). Each histological section was labeled by the neuropathologist. The labels covered four different infiltration grades (0% (healthy tissue), 0 to 30%, 30 to 60%, and >60% tumor infiltration), and various other structures like edema, necrosis, vessels, blood, connective tissue, coagulation, or cysts. The labels also differentiate whether the tumor infiltration or structure is located in white matter or gray matter (the cortex). The grade of tumor infiltration was selected subjectively based on the experience of the neuropathologist.

**Table 1:** Overview over the different patients and their diagnosis and the number of samples considered during the tissue analysis.

**Patient**	**Diagnosis**	**Number of Samples**
1	Oligodendroglioma—WHO II	6
2	Glioblastoma multiforme—WHO IV	7
3	Metastasis	4
4	Glioblastoma multiforme—WHO IV	6
5	Glioblastoma multiforme—WHO IV	5
6	Anaplastic astrocytoma—WHO III	3
7	Glioblastoma multiforme—WHO IV	5
8	Metastasis	3
9	Anaplastic oligodendroglioma—WHO III	6
10	Metastasis	5
11	Glioblastoma multiforme—WHO IV	4
12	Glioblastoma multiforme—WHO IV	5
13	Metastasis	2
14	Oligodendroglioma—WHO II	5
15	Glioblastoma multiforme—WHO IV	7

### 2.3 Data preparation

The preparation of the acquired data includes the transfer of the histological information onto the OCT B-scans and the creation of a dataset for the tissue classification. The transfer of the histological information onto the OCT B-scans will only be described briefly. A detailed description of the process can be found in (30). The first step was to find OCT B-scans that corresponded to the histological section. The cutting lines, which were used to determine the cutting position, were transferred onto the OCT volume by an image registration process. The field of view of the SD-OCT is known in the top-view white light image of the sample since both modalities were acquired by the same system. A registration between the two OCT volumes based on their topological height information, extracted from the tissue surface, enables the transformation of the cutting lines between the OCT volumes. The registration was performed by iteratively minimizing the dissimilarity between the topological height maps of the OCT volumes. The corresponding OCT B-scans were then extracted along the transferred cutting lines (**Figure 1E**). The transformation of the histological information onto the corresponding OCT B-scans was performed through a non-affine registration approach based on the tissue shape (**Figure 1F**). The tissue content was extracted from the histology and the corresponding OCT B-scan in the form of binary masks. This was possible because the tissue dimensions were known due to the tissue shaping by the agarose imprint. The non-affine transformation was then determined between the masks based on the shape of the tissue information using shape context features and a thin plate spline interpolation. Then, the determined transformation is then applied to the labeled image. As a result, 693 labeled OCT B-scans were created for each of the two systems.

**Figure 2:**
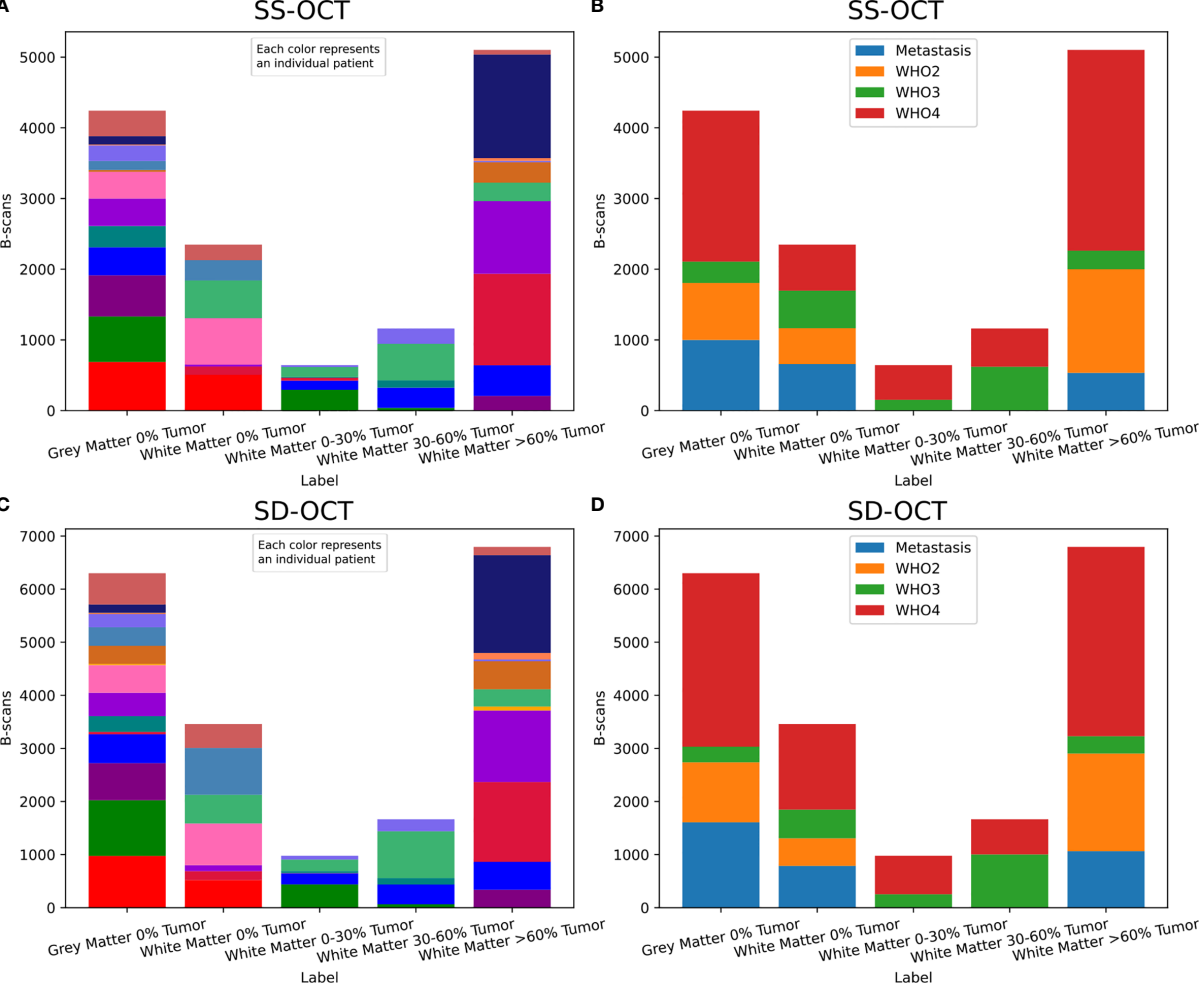
Distribution of the extracted B-scan patches among the different study patients [SS-OCT: **(A)**, SD-OCT: **(C)**] and the diagnosed tumor types [SS-OCT: **(B)**, SD-OCT: **(D)**]. For **(A)** and **(C)** the different patients were represented through different colors.

**Figure 3:**
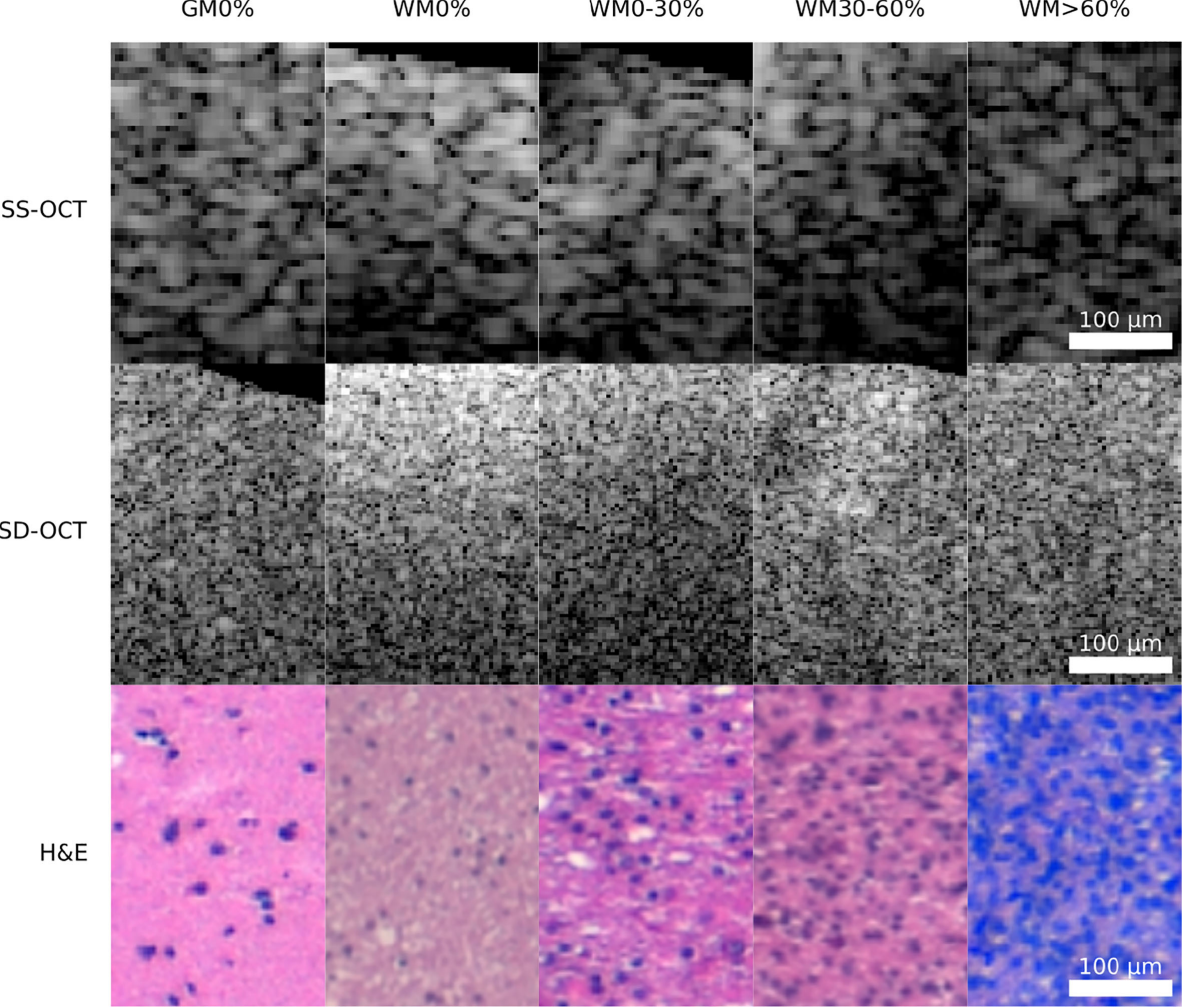
Example patches of each OCT-system for the five different tissue types (GM0%, gray matter 0% tumor infiltration; WM0%, white matter 0% tumor infiltration; WM0–30%, white matter with 0–30% tumor infiltration; WM30–60%, white matter with 30–60% tumor infiltration; WM >60%, white matter with >60% tumor infiltration).

From each B-scan, homogeneous patches with a size of around 300 × 200 μm^2^ (SS-OCT: 50 × 50 pixel; SD-OCT: 100 × 50 pixel) were extracted (**Figure 1G**). Each patch overlapped with the one before by 10 pixels and started 10 pixels below the tissue surface. This process resulted in two datasets, one for each OCT system, which were used for further processing. **Figure 2** shows the distribution of the labels among the different study patients and the diagnosed pathology. Deviations in the sample numbers of patients or tumor types for the two OCT systems were due to differences in the lateral pixel sizes and the differences in the field of view. **Figure 3** displays examples of B-scan patches.

### 2.4 Extracting optical parameters

The determination of optical properties from OCT data has already been used for tissue characterization by several groups (7, 24, 29, 31). Usually, the parameters used for the characterization of the tissue are the attenuation coefficient *μ* and the back-scattered intensity *I*_0_. Both parameters are influenced by the scattering anisotropy, which changes with the composition of the tissue (e.g., increasing tumor infiltration) (32, 33). The evaluation of those parameters for the two OCT systems with different wavelengths and optical set-up also allowed the comparison of possible differences in tissue classification accuracy for both settings. There are different models to retrieve optical parameters from OCT data (31). In this case, a single scattering model was chosen, which describes the exponential signal decay along an OCT A-scan. The model is given by the following:


(1) $$A^{2}(z)=r(z)\cdot h(z)\cdot I_{0}\exp(-2\mu z)$$

Here *A* ^2^(*z*) ∈ *ℝ* ^*L*^ was the measured intensity by the OCT system. *r* (*z*) is the intensity roll-off, which describes a system dependent on intensity decreasing along the depth. For the SD-OCT system roll-off is caused by the finite resolution of the spectrometer, which cannot properly resolve the high fringe frequencies in the depth (34–36). For the SS-OCT system, the roll-off was neglected since the SS-OCT does not use a spectrometer for signal detection. *r*(*z*) was given by (35):


(2) $$r(z)={\left(\frac{\sin(\xi )}{\xi }\right)}^{2}\cdot \exp\left(-\frac{\xi^{2}\omega^{2}}{2ln2}\right)$$

Here *ξ* is the imaging depth normalized to the maximum possible imaging depth, while *ω* is the relationship of wavelength spacing between pixels and the spectral resolution of the spectrometer. For the SD-OCT system, the roll-off was determined by measuring the signal of a mirror, which was moved through the imaging window by only adjusting the length of the reference arm. Equation (2) was then fitted to the maximum signal intensity of each measurement.

*h*(*z*) describes the light collection efficiency along the depth axis by the objective (14, 36), where *z*_*f*_ is the focus position, *z*_*r*_ the Rayleigh length, and *n* the refractive index of the imaged medium.


(3) $$h(z)=\frac{1}{1+{\left(\frac{z-z_{f}}{nz_{r}}\right)}^{2}}$$

**Figure 4:**
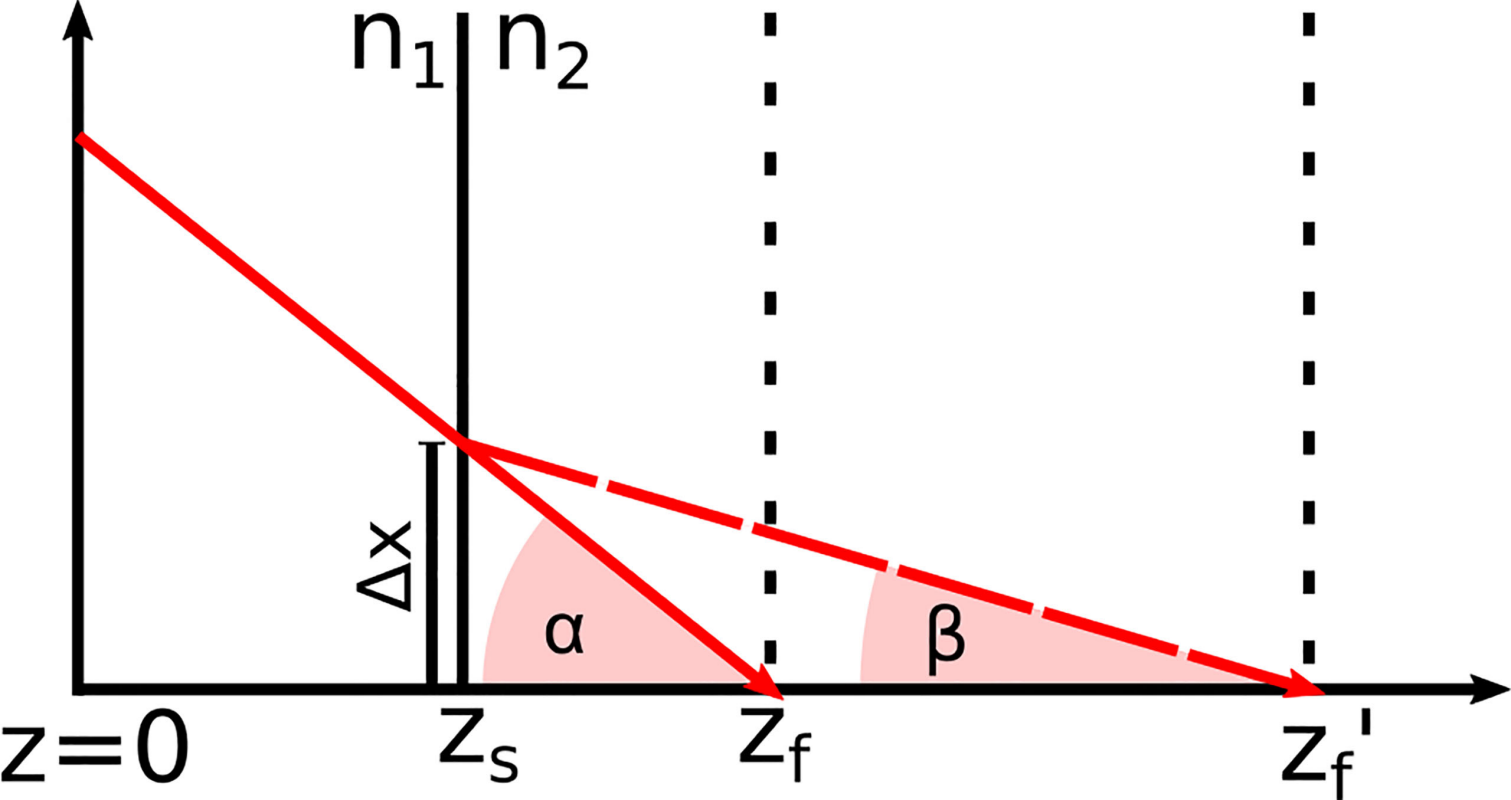
Refraction of the incident laser light at the tissue surface *z*_*s*_ due to the change of the refractive index from *n*_1_ to *n*_2_ This effect shifts the focus position *z*_*f*_ in air to *z*_*f*_′ in the medium.

*h*(*z*) was determined for both systems by moving a mirror through the imaging window while the reference length and the focus position were fixed. Equation (3) was fitted to the maximum signal intensity of each measurement after each measurement was corrected with *r*(*z*). For a medium with a refractive index *n*≠1, the focus position shift: Due to the difference in the refractive index between air and the medium, the refractive angle changes, which shifts the position of the focus *z*_*f*_′. Based on Snell’s law and simple geometrical considerations sketched in **Figure 4**, Equations (4) to (7) were derived. By setting Equations 6 and 7 equal, the correct focus position *z*_*f*_', is given by Equation (8).

Based on **Figure 4**, Equations (6) and (7) were derived. From these equations, Equation (8) was formed to calculate the corrected focus position. The angles *α* and *β* ere derived from Snell’s law of refraction and resulted in Equations (4) and (5).


(4) $$\alpha =\arcsin\;\left(\frac{NA}{n_{1}}\right)$$

(5) $$\beta =\arcsin\left(\frac{n_{1}\sin(\alpha )}{n_{2}}\right)$$

(6) $$\text{Δ}x=\tan(\alpha )\cdot (z_{f}-z_{s})$$

(7) $$\text{Δ}x=\text{tan}(\beta )\cdot (z_{f}\prime -z_{s})$$

(8) $$z_{f}\prime =\frac{\tan(\alpha )}{\tan(\beta )}\cdot (z_{f}-z_{s})+z_{s}$$

After the determination of *r*(*z*) and *h*(*z*), *A* ^2^(*z*) was corrected. Note, that *z* as assumed to be the geometrical length for *h*(*z*) and *A* ^2^(*z*), which means that the pixel size. Δ*s* was scaled with the refractive index of the medium present at that pixel *n*_*i*_ ( $z(i)=\sum_{i=0}^{L}\frac{\Delta s}{n(i)}$ ). In the case of this work *n* was either 1 for air or 1.36 for brain tissue (37–39). This resulted in an A-scan *A* ^2^(*z*)^′^, which only contains the signal decay caused by the tissue. The natural logarithm was applied to *A* ^2^(*z*)^′^, which brought the equation in a linear form. The optical parameters were then retrieved through a linear fit.


(9) $$\ln\;(A^{2}{\left(z\right)}^{\prime })=\text{ln}(I_{0})-2\mu z$$

For the application of the linear fit each B-scan patch was averaged to one A-scan. The fit was applied over a length of 300 μm. For the SD-OCT the length of the length corresponds to 100 pixel and for the SS-OCT 50 pixel.

### 2.5 Tumor classification

**Figure 5:**
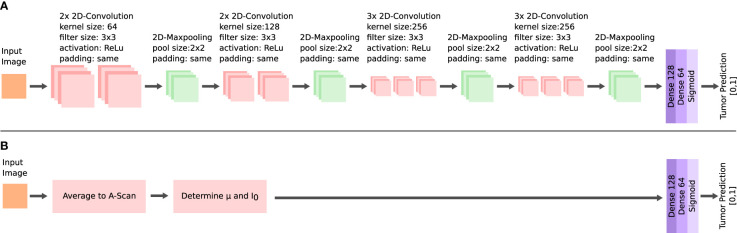
Architectures of the neural networks for the tumor classification. Feature extraction *via* multiple convolutional layers in the CNN **(A)** and extraction of the *μ* and *I*_0_ for the FCNN **(B)**.

A supervised classification was implemented for the dataset of each of the two OCT systems. The performance in this classification was used to evaluate the ability to identify the tumor infiltration zones. Four classification tasks were prepared for each acquired dataset. The complexity increased with each task. The first task (I) was the classification of healthy white matter from >60% infiltrated white matter. The second task (II) added the infiltrated data with 30 to 60% tumor to the dataset. For the third task (III), the data labeled with >030% tumor infiltrated white matter was added to the tumor data. The final task (IV) included the healthy gray matter data with the healthy white matter data. The classification was based on a convolutional neural network (CNN) (**Figure 5**). The network takes roll-off and focuses on corrected OCT B-scan patches as the input. The set-up allowed the neural network to consider optical and structural properties during the training. Information was extracted from each B-scan patch through four convolutional blocks. The structure of each block is similar to that of other image classification networks (23, 40). Each block consisted of two or three 2D-convolutional layers, with a filter size of 3, “same” padding, and “relu” activation. The number of filters per layer started at 64 and increased with each convolutional block. A 2D maxpooling layer with a size of 2 was applied at the end of each block, which down samples the information. The resulting feature map after the four convolutional blocks was flattened. The resulting feature vector was then put through two fully connected layers and a sigmoid layer, which outputs the predicted probabilities for each label.

For training, k-fold cross validation was applied (41). The training was repeated for each patient once as test data, while the remaining patients were used for the training data. To reduce errors due to the number of B-scan patches contributed by each sample, the training data were re-sampled for each training (see **Figure 2**). The B-scan patches of each under-represented sample were randomly duplicated to match the number of B-scan patches of the most prominent sample. Label imbalances were addressed in the same way, by randomly duplicating B-scan patches of each under-represented label to match the number of B-scan patches of the most prominent label. During the training, the data were randomly augmented by flipping and shifting the patches horizontally. For the optimization, an Adam algorithm with a learning rate of 0.001 and with a categorical cross-entropy function was chosen (42, 43). The batch size was set to 32 (44). The specificity and sensitivity were determined for the test set after the training for each fold (45). Each input was standardized with a zero mean.

An additional fully connected neural network (FCNN) was configured, which was used to better evaluate the performance of the CNN (**Figure 5**). The FCNN uses the attenuation coefficient*μ* and the backscattered intensity *I*_0_ as the input feature vectors, which were extracted from each B-scan patch as explained in the previous section. The FCNN architecture consists of two fully connected layers. The training configuration of the FCNN was the same as for the CNN. Both network approaches only differ in their method of extracting features from the OCT B-scan patches, which allowed the comparison of the different methods for feature extraction: The FCNN classification is based on optical parameters only, whereas the CNN classification can additionally consider structural information.

## 3 Results

### 3.1 Comparison of the optical properties

**Figure 6:**
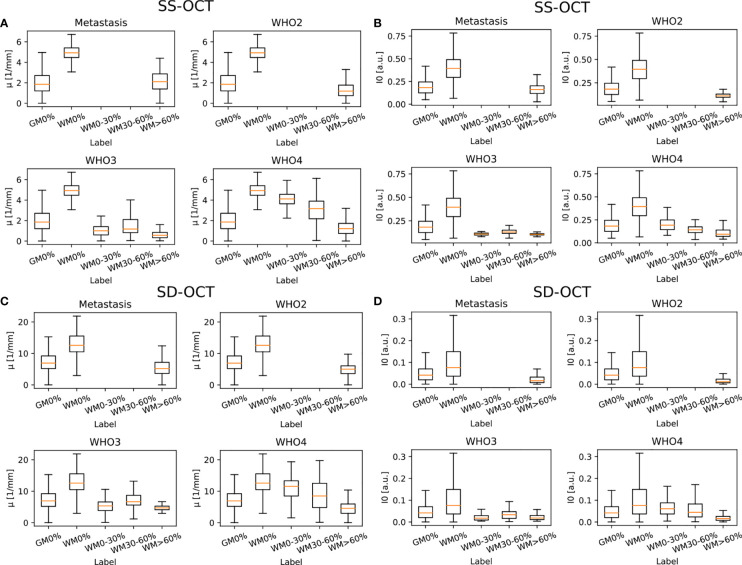
Determined optical properties *μ* (SS-OCT: **(A)** SD-OCT: **(C)** and *I*_0_ (SS-OCT: **(B)** SD-OCT: **(D)** for the four different pathologies (median value = orange line). Note that *I*_0_ was normalized to the maximum determined *I*_0_. The optical properties were determined for the following label: GM0%, gray matter 0% tumor infiltration; WM0%, white matter 0% tumor infiltration; WM0–30%, white matter with 0–30% tumor infiltration; WM30–60%, white matter with 30–60% tumor infiltration; and WM>60%, white matter with >60% tumor infiltration.

**Figure 6** shows the results for the attenuation coefficient *μ* and the backscattered intensity *I*_0_ determined from the averaged B-scan patches for each OCT system. Both parameters were determined separately for each pathology in order to better visualize possible differences. It is visible that, independent of the OCT system and the pathology, the median value of *μ* as well as *I*_0_ is lower in tumor infiltrated white matter than in healthy white matter. The optical properties of healthy gray matter differ from those of healthy white matter and are more similar to tumor infiltrated white matter.

**Table 2** shows the numerical values derived from **Figure 6** summarized for all pathologies. The presented data shows that the absolute values of the backscattered intensity and attenuation coefficient are different for the 930 nm and 1,300 nm OCT systems, but the relative differences in both values for different tissue types are similar. The relative differences were calculated for the two OCT systems and the optical properties for each possible tissue combination from **Table 2**. Afterwards, the median relative differences between the different systems were determined for each optical parameter. The median relative difference in the attenuation coefficient between the SD-OCT and the SS-OCT was 1.00[0.76;1.31]. A value of 1.00[0.64;1.54] was calculated for the maximum backscattered intensity

**Table 2:** Numerical values of the optical properties determined over all pathologies for the two OCT systems.

OCT-System	GM0%	WM0%	WM0–30%	WM30–60%	WM>60%
SD-OCT *μ* [mm−^1^]	6.93 [5.18;9.22]	12.58 [10.52;15.55]	10.21 [5.72;12.80]	6.95 [5.46;10.41]	4.79 [3.32;6.06]
SS-OCT *μ*. [mm−^1^]	1.85 [1.21;2.71]	4.93 [4.47;5.41]	3.91 [1.70;4.39]	2.05 [1.05;3.67]	1.22 [0.72;1.81]
SD-OCT *I*_0_ [a.u.]	0.04 [0.02;0.07]	0.08 [0.04;0.15]	0.05 [0.02;0.08]	0.04 [0.02;0.06]	0.01 [0.01;0.03]
-OCT *I*_0_ [a.u.]	0.18 [0.13;0.25]	0.40 [0.29;0.49]	0.16 [0.12;0.24]	0.13 [0.11;0.17]	0.11 [0.08;0.14]

The values presented are the median value and the 25th and 75th percentiles values respectively in brackets.

### 3.2 Results of tumor classification

**Table 3** displays the results for the different classification tasks specified in the section *Tumor classification*. The two neural networks delivered reasonable classification results for the classification tasks I to III independent of the OCT system. The two neural networks struggled with task IV, indicating a high similarity between tumor infiltrated white matter tissue and healthy gray matter, which was already visible when evaluating the optical parameters (section *Comparison of the optical properties*). For the SS-OCT, the performance of the CNN and FCNN was similar, although the CNN had the chance to extract structural features. The presence of structural features in B-scans of the SS-OCT could have been limited due to the low resolution compared to the SD-OCT. For the SD-OCT, a difference in the performance of the CNN and FCNN is visible for classification task I. This indicates, that for this case the CNN could extract additional features from the B-scans. This was expected since a higher resolution should have created B-scans with more details. For all other classification tasks, the performance of the two classification approaches was similar.

**Table 3:** Sensitivity and specificity for the different classification tasks, determined on the k-fold cross-validated test data from the SS-OCT and SD-OCT dataset and for both used neural network (CNN/FCNN).

OCT system	Neural network	Metric	I	II	III	IV
SS-OCT	CNN	Sensitivity	0.97 ± 0.05	0.89 ± 0.16	0.89 ± 0.14	0.58 ± 0.20
SS-OCT	CNN	Specificity	0.95 ± 0.06	0.86 ± 0.22	0.79 ± 0.29	0.63 ± 0.27
SS-OCT	FCNN	Sensitivity	0.92 ± 0.20	0.87 ± 0.20	0.84 ± 0.21	0.56 ± 0.34
SS-OCT	FCNN	Specificity	0.96 ± 0.07	0.94 ± 0.08	0.86 ± 0.24	0.62 ± 0.38
SD-OCT	CNN	Sensitivity	0.91 ± 0.14	0.85 ± 0.19	0.83 ± 0.19	0.54 ± 0.19
SD-OCT	CNN	Specificity	0.95 ± 0.03	0.76 ± 0.20	0.62 ± 0.13	0.65 ± 0.17
SD-OCT	FCNN	Sensitivity	0.81 ± 0.25	0.75 ± 0.24	0.72 ± 0.25	0.63 ± 0.26
SD-OCT	FCNN	Specificity	0.85 ± 0.06	0.81 ± 0.08	0.76 ± 0.10	0.57 ± 0.22

The four different classification tasks include the classification healthy white matter from >60% infiltrated white matter (I), classification healthy white matter from >30% infiltrated white matter (II), classification healthy white matter from >0% infiltrated white matter (III), and classification healthy white matter and gray matter from >0% infiltrated white matter (IV).

## 4 Discussion

Achieving full brain tumor removal during glioma and metastasis surgery increases the likelihood of the length of survival of patients and reduces the chance of tumor recurrence (2, 46). In the past, several other research groups have demonstrated that OCT might be a possible additional imaging method for detecting tumorous brain areas (7, 18–20, 23).

Regarding the determination of the optical properties, Yashin et al. and Kut et al. calculated the attenuation coefficients for gray matter, white matter, and different degrees of tumor infiltration for different tumor types and used a similar imaging wavelength of 1,300 nm for the data acquisition, the same as that of the SS-OCT used in this work (7, 24). Yashin et al. determined, among others, the attenuation coefficients for white matter (8.5 mm^−1^), gray matter (2.5 mm^−1^), and glioblastoma multiforme (3.0 to 5.5 mm^−1^) (24). Kut et al. provided the attenuation coefficients for white matter and different tumor infiltration grades: healthy white matter (6.2 ± 0.8 mm^−1^), tumor infiltrated white matter (3.5 ± 0.8 mm^−1^), and tumor core (3.9 ± 1.6 mm^−1^) (7). When comparing the results of these groups with the presented results of this work, it stands out that the absolute values differ, but the relative trends are the same. The decrease in the attenuation coefficient in white matter with increasing tumor infiltration is clearly visible and was explained by Yashin et al. with the degradation of the myelin fibers, which are present in healthy white matter (24, 47). The lack of myelin fibers aggravates the separation of gray matter from tumor-infiltrated white matter because the attenuation values for gray matter overlap with tumor-infiltrated white matter. The differences in the absolute attenuation values among the research groups can be caused by different properties of the OCT systems or different approaches in the signal processing. For example, Kut et al. used a reference phantom in order to compensate for focus and roll-off effects (7). Yashin et al. did not consider these effects, which lead to higher signal decay and therefore higher attenuation values (24). Another reason could be the freshness of the imaged *ex vivo* samples. Kiseleva et al. showed that the attenuation coefficient can vary significantly between *in vivo* and *ex vivo* samples (29). They determined smaller attenuation coefficients for *in vivo* than for *ex vivo* samples. It was also demonstrated that the attenuation coefficients change with increasing time after extraction of the sample (29). The sample processing of this work was done within 15 min, while Yashin et al. processed the samples after 15 to 30 min (19, 24). Kut et al. did not disclose the age of the *ex vivo* samples (7). Therefore, it is possible that the fresher samples used in this work lead to lower attenuation coefficients.

The determined attenuation coefficients for the SD-OCT with an imaging wavelength of 930 nm cannot be compared with other groups because research groups using an imaging wavelength of around 930 nm did tumor classification based on structural information (20, 21) or conducted a qualitative analysis (48). According to Almasian et al., the attenuation coefficient derived from an OCT A-scan should be smaller than the scattering coefficient stated in the literature (49) because in tissues with high scattering properties, multi-scattered light can influence the OCT signal (50). This is the case for the determined values. The higher attenuation values compared to 1,300 nm were expected because the scattering in brain tissue increases with decreasing imaging wavelength (49). The relative trends visible in **Figure 6** have the same explanation as for the attenuation values of the SS-OCT.

Regarding tumor classification based on the convolutional neural network, different stages of tumor discrimination were shown. Each classification task resembled the classification problems raised by other research groups. The most popular one is the tumor classification between healthy white matter and highly tumor-infiltrated tissue. For both datasets, very good classification results were achieved because the optical properties of healthy white matter and highly tumor-infiltrated white matter are very different. The results achieved in this task are comparable with those of other groups that used machine learning-based classification approaches. For example, Juarez-Chambi et al. achieved a sensitivity of 99% and a specificity of 86% with an A-scan based approach. Other groups used B-scan based approaches and achieved classification accuracies above 93% for very structural tumors like meningioma, metastasis, or highly infiltrated white matter (20, 21, 23). In their study, Gesperger et al. raised the problem that the classification accuracy is reduced significantly if tumor-infiltrated tissue is not considered during the training. Classification tasks II and III considered tumor infiltration zones during the training, which led to a significant decrease in the classification specificity. This seems plausible, since the properties of less infiltrated regions can be very similar to those of healthy white matter, which can lead to false classifications. The fourth classification task added healthy gray matter to the classification data. Since the optical properties are very similar to those of tumor-infiltrated tissue, the neural network could not extract features from the B-scans to achieve a good classification result. In contrast to this work, Lenz et al. demonstrated that discrimination of healthy gray matter and white matter from meningioma is possible if structural information (e.g., Haralick’s texture features) is considered. This suggests that more features need to be considered for this classification task.

For all classification tasks, the approach using only the optical parameters, attenuation coefficient, and backscattered intensity achieved similar results for all classification tasks as the approach using the convolutional neural network. This shows that the optical parameters are strong descriptive features, which is not surprising because other groups achieved good classification results using only optical features (7, 24). It also gives the impression that the structural information in the extracted B-scan patches was not significant enough to improve the classification results for the SS-OCT. The reason for this result could be the low resolution of the OCT-system, which is not high enough to make structural differences visible. On the other hand, the SD-OCT showed a difference in the classification performance for the classification of healthy white matter and white matter with a tumor infiltration of >60%. Here, the CNN could extract more information from the OCT B-scan patches in improve the classification result, which could be a result of the higher resolution of the SD-OCT.

Comparing the OCT systems showed that both of them performed very similarly, regarding the determination of the optical properties and discrimination of tumor from healthy tissue. However, the results of the SD-OCT show slightly higher fluctuations in the measured optical properties than the SS-OCT. This could be a result of the higher resolution. Due to the higher detail of the OCT B-scans, small tissue variations, like blood accumulations created through the excision or small air bubbles created through the embedding process, could create artefacts. These artefacts could be present in each B-scan and are hard to detect. They also aggravate the classification and determination of the optical properties. For B-scans acquired by the SS-OCT, these artefacts were already averaged out or were suppressed by the lower resolution, which led to more stable values in the classification and determined optical parameters. The higher numerical aperture of the SD-OCT was also a potential error source, since a higher numerical aperture leads to faster signal degradation with increasing distance from the focus position. This aggravates the compensation of the focus effects, which could lead to potential errors. Another limiting factor in this work was the limited size of the dataset. The small number of patients leads to unfavorable training and test combinations. These combinations resulted in bad classifications, which might not happen if the number of patients and therefore the number of samples was higher. A much higher number of patients and samples with low tumor infiltration is needed to allow a neural network to identify differences between healthy and tumorous tissue.

## 5 Conclusion

In conclusion, this work showed that OCT systems with different optical properties achieve similar results regarding the identification of brain tumors. The attenuation coefficient and the backscattered intensity of different tumor types were determined for each OCT system. The determined optical properties showed similar relationships between different tissue types independent of the OCT system. Based on B-scans and optical properties, different neural networks were trained for different classification tasks, which can occur during tumor resection. Both OCT systems achieved good classification results for separating healthy white matter from tumor-infiltrated white matter. Both OCT systems failed when it came to the discrimination of healthy gray and white matter from tumor infiltrated white matter because the optical properties of gray matter are similar to those of tumorous white matter. The achieved results will be used as a first benchmark in the future to test different approaches to the classification. The target will not only be to differentiate healthy white matter from tumor-infiltrated tissue but also to achieve good classification results even when gray matter or other tissue types are present in the dataset. More data needs to be collected in the future to cover most of the tissue combinations that can happen during tumor resection. Additionally, it was recently demonstrated by Theisen-Kunde et al. that the SS-OCT can be mounted to a surgical microscope, enabling *in vivo* real-time imaging of the brain during tumor resection (25). The methods presented in this manuscript will be applied to the *in vivo* data of that OCT system in order to progress towards the goal of guiding the neurosurgeon during tumor resection with the help of OCT.

## Data availability statement

The raw data supporting the conclusions of this article will be made available by the authors, without undue reservation.

## Ethics statement

The evaluation of human brain tissue was approved by the Ethics Committee of University Medical Center Schleswig-Holstein, Campus Luebeck, Germany, No.: 18-204. The patients/participants provided their written informed consent to participate in this study.

## Author contributions

Data acquisition and sample preparation: PS, BL, CG, WD, VD, SS-H, DT-K, CH, and MMB. Methology: PS and BL. Writing—original draft: PS. Review and editing: PS, BL, CG, WD, VD, SS-H, DT-K, CH, MB, HH, RH, and RB. All authors contributed to the article and approved the submitted version.

## Funding

This research is funded by the Federal Ministry of Education and Research Grant Nos. 13GW0227A, 13GW0227B, and 13GW0227C and the European Union project ENCOMOLE-2i (Horizon 2020, ERC CoGno. 646669). We acknowledge financial support by Land Schleswig-Holstein within the funding programme Open Access Publikationsfonds.

## Conflict of interest

WD and RH hold shares of Optores GmbH. RH receives royalties from patents licenced to Optores GmbH.

The remaining authors declare that the research was conducted in the absence of any commercial or financial relationships that could be construed as a potential conflict of interest.

## Publisher’s note

All claims expressed in this article are solely those of the authors and do not necessarily represent those of their affiliated organizations, or those of the publisher, the editors and the reviewers. Any product that may be evaluated in this article, or claim that may be made by its manufacturer, is not guaranteed or endorsed by the publisher.
